# Effects of the upright body type exercise program on foot balance in female high school students

**DOI:** 10.1186/1757-1146-7-S1-A135

**Published:** 2014-04-08

**Authors:** Nam-Young Son, Joong-Sook Lee, Jeong-Ok Yang, Bom-Jin Lee, Dong-Wook Han

**Affiliations:** 1 Department of Physical Education, College of Medical and Life Sciences, Silla University, Busan, Korea; 2 Department of Physical Therapy, College of Medical and Life Sciences, Silla University, Busan, Korea

## Background

The foot balance is strongly associated with the body posture which in turn, contributes to musculoskeletal functioning. Unfortunately, children and adolescents in these day often encounter some problems with spinal alignment such as scoliosis, due to the sedentary lifestyle. Conversely, An exercise program specially designed for body posture and balance may be the key to solve these programs. However, not much research has been conducted to determine the benefits of exercise in relation to correct the body posture, as well as foot balance.

## Materials and methods

The purpose of this study was to investigate the effects of an upright body-type exercise program on the foot balance in female high school students. Forteen female high school students were selected and grouped into an experiment(n=7) and control(n=7) group. The research varibles included foot balance and body posture which were measured by Shesei Innovation System (PA 200, Japan). A specially designed exercise program called the upright body-type exercise was developed and implemented for 12 weeks (2 times per week).

## Results

**Figure 1:**
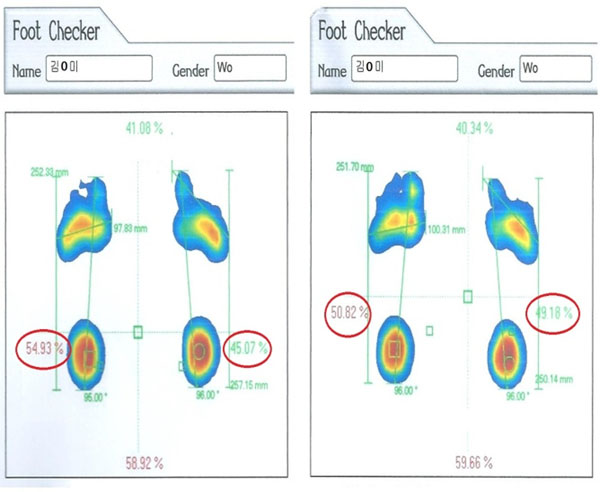
An example of changes in the foot balance of participant 1.

**Figure 2:**
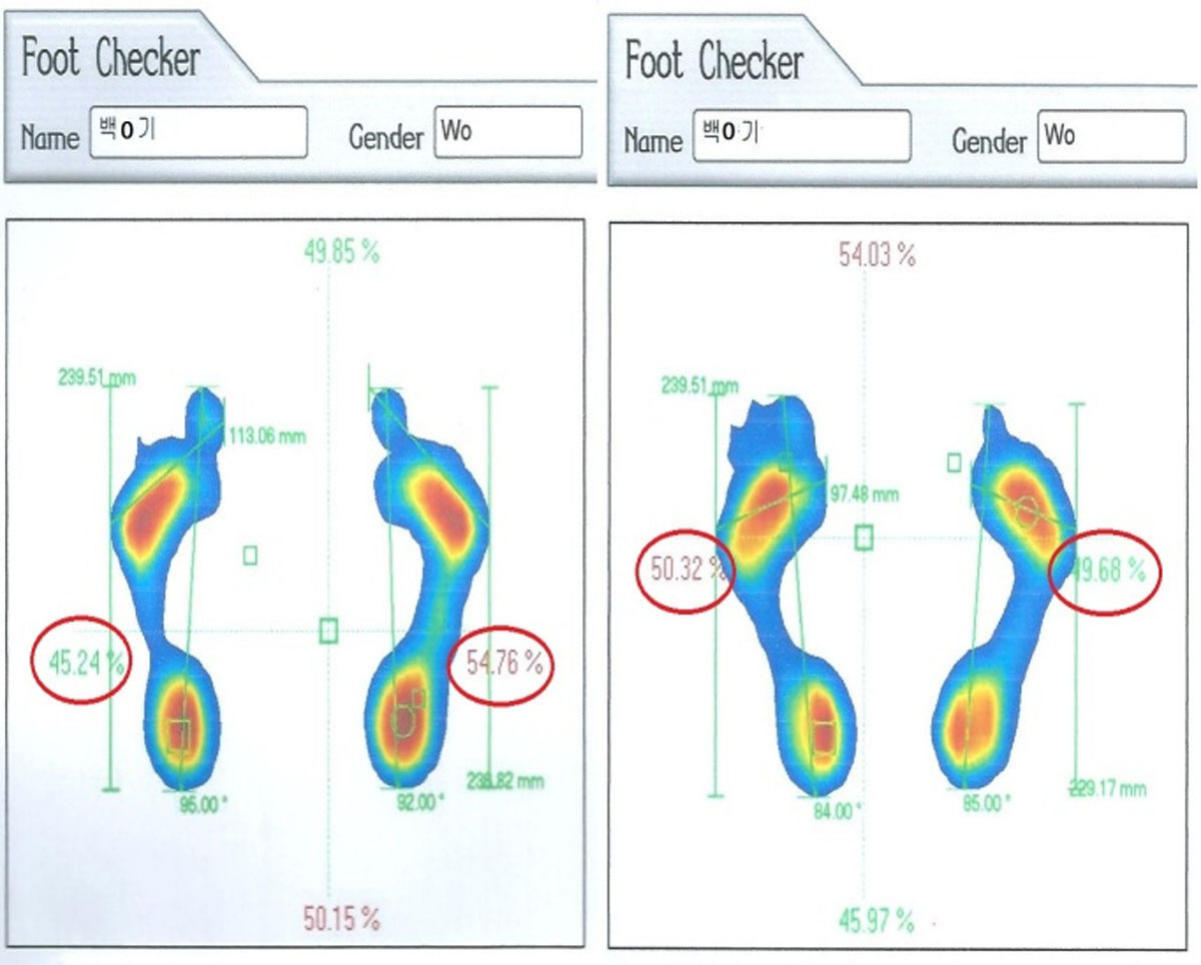
An example of changes in the foot balance of participant 3.

Results revealed that the left balance was changed to almost the perfect balance (50%) from 48.93±3.87 to 48.97±2.95; whreas, the right foot balance was from 51.07±3.87 to 50.26±2.95 in experimental group which were also near to the perfect balance (50%). However, the mean score of the left foot balance in the control group was decreased from 49.97±2.67 to 49.08±1.41; whereas, that of the right foot balance was somewhat increaed from 50.03±2.67 to 50.92±1.41. Figures 1 and 2.

## Conclusions

As a conclusion, the upright body-type exercise program may have positive impact on the foot balance and body posture in female high school students. This program may also be utilized for people with spinal conditions, as a means of rehabilitation.
